# Determinants of change in timely first antenatal booking among pregnant women in Ethiopia: A decomposition analysis

**DOI:** 10.1371/journal.pone.0251847

**Published:** 2021-06-04

**Authors:** Melaku Yalew, Bezawit Adane, Yitayish Damtie, Bereket Kefale, Reta Dewau, Kefale Mektu, Mastewal Arefaynie, Assefa Andargie

**Affiliations:** 1 Department of Reproductive and Family Health, School of Public Health, College of Medicine and Health Sciences, Wollo University, Dessie, Ethiopia; 2 Department of Epidemiology and Biostatistics, School of Public Health, College of Medicine and Health Sciences, Wollo University, Dessie, Ethiopia; 3 Department of Physiology, School of Medicine, College of Medicine and Health Sciences, Wollo University, Dessie, Ethiopia; Universitas Airlangga, INDONESIA

## Abstract

**Background:**

Even though maternal health was highly targeted in different global strategies, maternal mortality could not be decreased as was expected. Besides this, prior decomposition analysis to the possible cause of changes to timely first antenatal booking has not been conducted. Therefore, this study aimed to assess determinants of change in timely first antenatal booking among pregnant women in Ethiopia.

**Methods:**

The study utilized three consecutive Ethiopia Demographic and Health Survey (EDHS) datasets which were collected through cross-sectional study design. The number of pregnant women who gave birth in 2005, 2011 and 2016 survey included in the analysis was 7,307, 7,908 and 7,590 respectively. The data were analyzed by using Stata/SE version 14.0. Logit-based decomposition analysis was done to identify contributing factors for change in timely first antenatal booking and statistical significance was determined by using P-value.

**Results:**

The trend of timely first antenatal booking was increased from 6% to 20% in the last ten years. The analysis revealed that 14% of the overall change was because of the change in women’s composition. Changes in the composition of women according to region, education and occupation status were the major sources of this change. The remaining, 86% of the change was due to differences in the coefficient. Mostly, the change in behaviors of the Oromia population, those who have lived in the rural areas and male household head were some of the contributing factors for the increase in timely first antenatal booking.

**Conclusions:**

There was a significant increase in timely first antenatal booking among pregnant women in Ethiopia from 2005 to 2016 EDHS. The change in the women composition according to residency, education and occupation status of women and the difference in behaviors like: behavior of rural residents and male household head contributed to the majority of the change. Interventions targeting the male household head, rural residents and those women who lived in the Oromia region should be emphasized to increase further timely booking. In addition, promoting women in terms of education and creating job opportunities could be the other recalled intervention areas of the country.

## Introduction

Even though maternal mortality rate was decreased by 45% from 1990 to 2013, complications during pregnancy and childbirth are the leading causes of death and disability in reproductive age women, especially in developing countries [1, 2]. According to the World Health Organization (WHO), 275 to 288 maternal deaths were reported due to pregnancy and related complications [3]. The burden is very high in developing countries accounting for 99% of global deaths and Sub-Saharan African countries including Ethiopia shares 66% of the mortality [4].

In response to this, antenatal care (ANC) program was introduced by WHO at the beginning of 21 century as a constellation of different services and interventions that a pregnant woman should receive from skilled health care providers [5]. It aims to prevent and identify pregnancy risks and treat complications timely by providing appropriate information [6–8]. Under normal conditions, at least four antenatal visits are required and the first visit should take place within the first trimester of pregnancy [9, 10].

Despite its importance, only 24% of pregnant women in low income countries received ANC as compared to higher income countries (82%) [11]. Not only had this but also timely initiating ANC is very important to early detecting pregnancy-related complications and adverse birth outcomes. However, most of pregnant women initiated ANC lately [12, 13]. The global coverage indicated that only 58.6% of women had early antenatal visits [11] and according to Demographic and Health Surveys (DHS) of 30 Sub-Saharan African countries, first trimester ANC initiation was only 37% [14]. Studies conducted in Zambia, Tanzania and Rwanda indicated that timely initiation first ANC was 28%, 29.6% and 39.9% respectively [15–17]. Similarly, the 2016 Ethiopia Demographic and Health Survey indicated that only 20% of women timely initiated their first ANC [18].

For those who missed ANC or initiated lately, the information and interventions about a woman’s reproductive health and the health of her newborn are also missed [19]. A study indicated that late ANC booking was linked to increased morbidity of mother and many maternal deaths were reported in women who didn’t initiate ANC timely [20–22]. Sub-optimal ANC was also found to be the major contributing factor for stillbirth [23, 24]. Not only this, but ANC affects appropriate infant feeding [25] and newborn care during postpartum period [26].

Different evidences showed that the timely first ANC booking was influenced by different factors like: socio-demographic factors (age, education, residence, occupation, income) [16, 27–30], age at marriage [31], wantedness of pregnancy [17, 32–36], no of living children [17], Parity [28, 37–40], distance to a health facility [17, 29, 41, 42]. Even though timely initiating first ANC was previously studied in different parts of Ethiopia, those studies were only localized to certain settings and their sample size was too small. So, the finding of those studies was less precise. Besides this, decomposition analysis helps to break down the discrepancies into its driving factors, thereby estimating the independent contribution of each of the factors to the observed differences in timely first antenatal booking between consecutive EDHS’s. The degree of the change and the contribution of the driving factors to these differences have not yet been studied in Ethiopia. That will help policymakers to design strategies to improve maternal and child health”. So, this study aimed to assess determinants of timely first antenatal booking among pregnant women in Ethiopia by using 2005, 2011 and 2016 Demographic and Health Survey data.

## Methods

### Data source, study area and study design

The study was conducted cross-sectionally in Ethiopia and it utilized the national surveys of 2005, 2011 and 2016 EDHS datasets. It was collected by the Central Statistical Agency (CSA) in coordination with the Federal Minister of Health (FMoH) and the Ethiopia Public Health Institute (EPHI) from April to August 2005, December 2010 to June 2011 January to June 2016 for the survey of 2005, 2011 and 2016 respectively. All EDHS datasets were accessed from measuredhs after requesting it through the web site www.measuredhs.com.

### Study population and variable measurement

**Fig 1:**
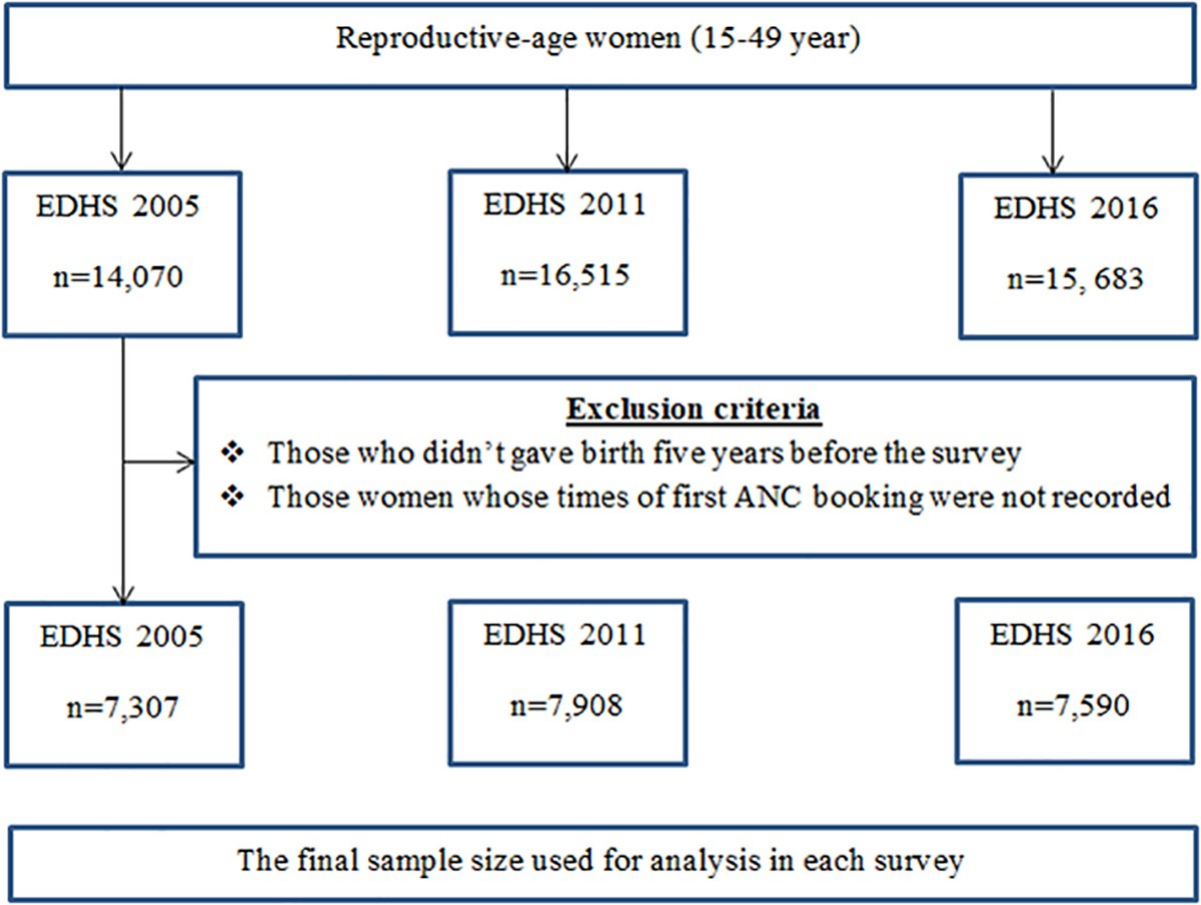
Sampling and exclusion procedures to identify the final sample size in 2005, 2011 and 2016 EDHS.

The data were only restricted to pregnant women who gave birth five years before the survey and their time of first antenatal booking was recorded. The sample sizes for each survey were 7,307, 7,908 and 7,590 women who gave birth five years before the survey in 2005, 2011 and 2016 EDHS respectively (Fig 1).

The outcome variable was classified dichotomously as “YES/NO”. A pregnant woman who gave birth five years before the survey was considered as she had timely first antenatal booking “YES” if she initiated her first antenatal visit before 12 completed gestational weeks, otherwise, (if she didn’t have at all or initiated later) considered as “NO” [31, 43–45]. The main independent factors were socio-demographic factors (age was categorized as ordinal variable as (“15–19”,”20–24”,”25–29”,”30–34”,”35–39”,”40–44”and”45–49”), education level also measured as ordinal categorical variable (“no education”, “primary education”, “secondary education” and “higher and above”), place of residence was measured as nominal categorical variable (“rural” and “urban”), age at first marriage was measured as ordinal variable (“less than 18nyears” and “18 years and above”), marital status was measured as nominal categorical variable (“married” and “not married”), sex of household head was measured as nominal categorical variable(“male” and “female”), wealth index was measured as ordinal categorical variable (“poorest”, “poorer”, “middle”, “richer” and “richest”), occupation measured as nominal categorical variable (“not working” and “working”), type of pregnancy was measured as nominal categorical variable (“wanted” and “not wanted”), parity was classified as null-Para or multi-Para and number of living children was measured as ordinal variable (“no children”, “1–5 children” and “6 or more children”).

### Data quality control and analysis

An initial exploratory data analysis was conducted to check for outliers, missing and inconsistency. All the results of this study were weighted for sampling probabilities using the weighting factor in the EDHS data. The complex sampling procedure was also considered by using the SVY STATA command to control the clustering effect of complex sampling. The data were analyzed by using Stata/SE version 14.0. Multivariate decomposition analysis was done to see determinants of change in timely first antenatal booking among pregnant women. The purpose of decomposition analysis was to identify the source of change in the last decade (2005 to 2016). As the changes in population composition and behavior of the population related to timely first antenatal booking were considered, the result focused on how timely first antenatal booking responded to population composition and their behavior and how these factors shape it across different surveys at different times. The analysis used the output for the logistic regression model to decompose the observed difference into two components. Hence, the observed differences in timely first antenatal booking between different surveys were decomposed into characteristics (natural endowment) and a coefficient (effect of characteristics). The logit based difference can be decomposed as [46].


$$Y=F(\frac{e^{X\beta }}{1+e^{X\beta }})$$ (1)

$$Ya-Yb=F\left(\frac{e^{Xa\beta a}}{1+e^{Xa\beta a}}\right)-F\left(\frac{e^{Xb\beta b}}{1+e^{Xb\beta b}}\right)$$ (2)

$$\Delta Y=\left[F(\frac{e^{Xa\beta a}}{1+e^{Xa\beta a}})-F(\frac{e^{Xb\beta a}}{1+e^{Xb\beta a}})\right]+\left[F(\frac{e^{Xb\beta a}}{1+e^{Xb\beta a}})-F(\frac{e^{Xb\beta b}}{1+e^{Xb\beta b}})\right]$$ (3)

Where: Y is the dependent variable, X is the independent variable, **β** is the coefficient and F is differential logistic function of $X\left(\frac{e^{X\beta }}{1+e^{X\beta }}\right)\;and\;Y.$ So, the result focused on how timely first antenatal booking responded to population composition and their behavior and how these factors shape it across different surveys at different times. Statistical significance was determined at a P value of less than 0.05.

### Ethical considerations

The data were accessed from DHS by requesting it through a web site: www.measuredhs.com. Then, authorization letter was also received to use EDHS dataset. The data were used only for this study and it was not passed to other researchers. All data were treated as confidential and no personal or household identifiers were used in the survey.

## Results

### Characteristics of the respondents

Table 1 represents the characteristics of study participants over the three consecutive EDHS and a total of 7,307, 7,908 and 7,590 pregnant women who gave birth five years before the survey were included in the analysis in 2005, 2011 and 2016 respectively. Among the respondents, the proportions of women whose ages between 35–39 years were constant in all three surveys. Whereas, the percentages of women who got their first marriage less than 18 years were gradually decreased from 70% in 2005 to 63% in 2016. Similarly, women who were not educated found to be decreased from 78% to 63% in the last 10 years. However, the proportions of women who lived in the Oromia region were increased in the subsequent survey (37% in 2005, 39% in 2011 and 41% in 2016). Concerning the religion of participants, about 45%, 42% and 38% of them were Orthodox followers in 2005, 2011 and 2016 EDHS respectively. The proportions of women who had unwanted childbirth were decreased from 2005 to 2016 EDHS (37.08% to 26.57%). The mean total numbers of living children and the number of children ever born were almost similar across all surveys which were 1.2 and 1.8 respectively (Table 1).

**Table 1:** Percent distribution of characteristics of participants, in 2005, 2011 and 2016 Ethiopia Demographic and Health Surveys.

Characteristics	EDHS 2005	EDHS 2011	EDHS 2016
	n = 7,307	n = 7,908	n = 7,590
**Age of women**			
15–19	6.02	5.08	4.47
20–24	20.16	20.34	19.29
25–29	26.83	30.13	28.53
30–34	19.54	18.83	21.89
35–39	15.57	15.67	15.89
40–44	7.92	7.22	7.20
45–49	3.96	2.73	2.73
**Age at first marriage**			
<18	70.37	66.02	62.63
≥ 18	29.63	33.98	37.37
**Education status of women**			
Not educated	78.47	66.64	63.13
Primary	16.49	28.71	28.32
Secondary	4.49	2.85	5.53
Higher and +	0.55	1.80	3.02
**Marital status**			
Not married	7.32	9.14	6.34
Married	92.68	90.86	93.66
**Place of residence**			
Urban	8.67	15.02	12.77
Rural	91.33	84.98	82.23
**Occupation status of women**			
Not working	69.14	45.34	55.25
Working	30.86	54.66	44.75
**Religion**			
Orthodox	44.64	42.08	37.97
Protestant	19.21	22.29	21.76
Muslim	32.60	32.41	37.21
Others	3.55	3.22	3.06
**Region**			
Tigray	6.57	6.70	7.08
Afar	0.93	0.99	0.94
Amhara	25.39	25.18	21.50
Oromia	37.27	39.41	41.24
Somali	3.95	2.50	3.54
Benishangul Gumuz	0.94	1.17	1.06
SNNP	22.33	20.66	21.09
Gambela	0.32	0.38	0.27
Harari	0.20	0.24	0.23
Addis Ababa	1.76	2.44	2.61
Dire Dawa	0.34	0.33	0.44
**Wealth index**			
Poorest	20.80	21.99	21.76
Poorer	21.26	21.45	21.79
Middle	21.71	20.59	20.93
Richer	19.86	18.89	18.80
Richest	16.37	17.08	16.72
**Sex of household head**			
Male	87.85	83.60	85.29
Female	12.15	16.40	14.71
**Distance to a health facility**			
Big problem	73.23	72.91	58.06
Not a big problem	26.77	27.09	41.94
**Pregnancy wanted**			
No	37.08	32.28	26.57
Yes	62.92	67.72	73.43
**Parity**			
Null para	16.29	17.69	18.90
Multi para	83.71	82.31	81.10
**Number of living children**			
0	1.72	1.01	0.65
1–5	76.85	79.80	77.55
≥ 6	21.43	19.19	21.80

Others = Catholic and traditional followers

### Trends of first antenatal booking

**Fig 2:**
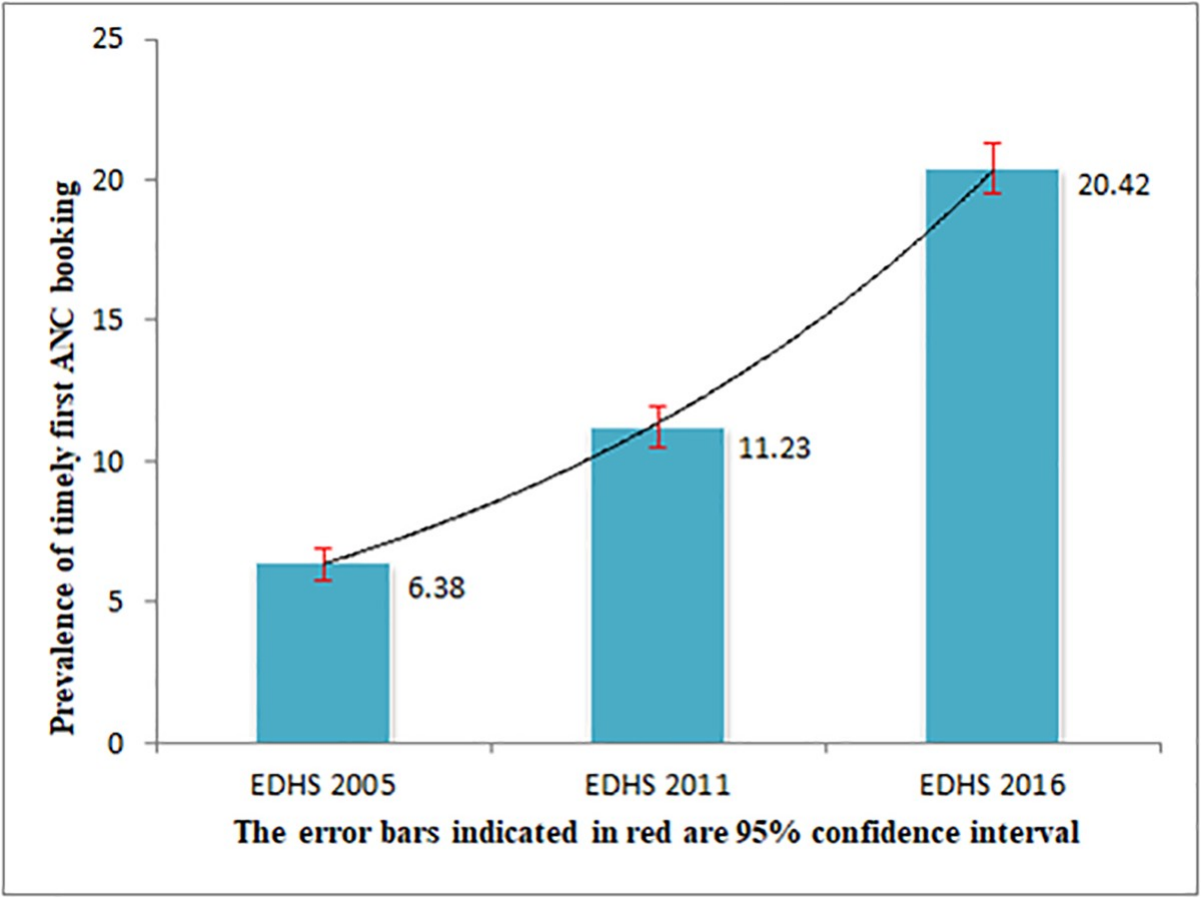
The trend of timely first antenatal booking among pregnant women in Ethiopia (EDHS 2005 to 2016).

The overall trend of timely first antenatal booking among pregnant women in Ethiopia was significantly increased, from 6.38% in 2005 to 20.42% in 2016 (Fig 2).

The trend of timely first antenatal booking among pregnant women showed variation per different respondents’ characteristics. It was increased in all age groups and the highest increment was 22.36% and the lowest was 10.99% in the age of 15–19 and 35–39 years respectively. The trend of timely first antenatal booking was greatly increased in rural dwellers as compared to urban. Similarly, first antenatal booking was increased across all levels of education and the highest increment was observed in study participants who were primarily educated as compared to others. In four regions; Tigray, Amhara, Gambela and Harari and one administrative town (Dire Dawa), the change in first antenatal booking among pregnant women showed an increment above 20%. It was also increased by 20% in null-para and above 30% in women not having living children (Table 2).

**Table 2:** The trend of timely first antenatal care booking among pregnant women in Ethiopia by characteristics of respondents, 2005, 2011 and 2016 EDHS (%).

Characteristics	EDHS 2005	EDHS 2011	EDHS 2016	Percentage point difference in 1^st^ ANC		
	n = 7,307	n = 7,908	n = 7,590	Phase І 2011–2005	Phase ІІ 2016–2011	Phase ІІІ 2016–2005
**Age of women**						
15–19	4.67	9.76	27.03	5.09	17.27	22.36
20–24	8.32	11.74	21.23	3.42	9.49	12.91
25–29	7.46	12.2	24.2	4.74	12	16.74
30–34	6.2	11.18	18.45	4.98	7.27	12.25
35–39	4.85	11.47	15.84	6.62	4.37	10.99
40–44	4.48	8.81	16.74	4.33	7.93	12.26
45–49	2.62	4.96	16.51	2.34	11.55	13.89
**Age at first marriage**						
<18	5.41	10.2	19.64	4.79	9.44	14.23
≥18	8.49	13.11	21.55	4.62	8.44	13.06
**Education status of women**						
Not educated	3.92	7.31	15.38	3.39	8.07	11.46
Primary	9.21	15.64	24.61	6.43	8.97	15.4
Secondary	34.5	34.51	40.04	0.01	5.53	5.54
Higher and +	44.38	49.46	50.56	5.08	1.1	6.18
**Marital status**						
Not married	9.9	14.66	24.61	4.76	9.95	14.71
married	6.11	10.89	20.14	4.78	9.25	14.03
**Place of residence**						
Urban	32.44	30.99	44.07	-1.45	13.08	11.63
Rural	3.91	7.74	16.96	3.83	9.22	13.05
**Region**						
Tigray	7.46	15.51	33.04	8.05	17.53	25.58
Afar	4.39	10.84	18.87	6.45	8.03	14.48
Amhara	5.89	10.46	27.77	4.57	17.31	21.88
Oromia	6.17	9.49	14.75	3.32	5.26	8.58
Somali	3.92	9.2	13.73	5.28	4.53	9.81
BenishangulGumz	4.38	6.24	15.9	1.86	9.66	11.52
SNNP	4.04	9.22	15.06	5.18	5.84	11.02
Gambela	10.32	24.34	32.03	14.02	7.69	21.71
Harari	18.32	32.64	44.7	14.32	12.06	26.38
Addis Ababa	44.9	49.94	59.93	5.04	9.99	15.03
Dire Dawa	29.02	35.01	60.33	5.99	25.32	31.31
**Religion**						
Orthodox	8.86	15.27	25.57	6.41	10.3	16.71
Protestant	3.72	7.41	12.53	3.69	5.12	8.81
Muslim	5.03	8.81	18.31	3.78	9.5	13.28
Others	2.07	9.37	13.4	7.3	4.03	11.33
**Occupation status of women**						
Not working	5.67	10.42	17.89	4.75	7.47	12.22
Working	7.98	11.92	23.55	3.94	11.63	15.57
**Wealth index of household**						
Poorest	2.07	5.01	14.24	2.94	9.23	12.17
Poorer	2.58	6.49	15.42	3.91	8.93	12.84
Middle	4.56	7.13	18.6	2.57	11.47	14.04
Richer	4.42	10.94	19.64	6.52	8.7	15.22
Richest	21.61	30.45	38.12	8.84	7.67	16.51
**Sex of household head**						
Male	5.51	10.36	19.79	4.85	9.43	14.28
Female	12.69	15.67	24.08	2.98	8.41	11.39
**Distance to health facility**						
Big problem	4.89	8.51	15.84	3.62	7.33	10.95
Not big problem	10.46	18.57	26.76	8.11	8.19	16.3
**Pregnancy wanted**						
No	6.92	11.89	17.64	4.97	5.75	10.72
Yes	6.07	10.92	21.42	4.85	10.5	15.35
**Parity**						
Null-para	10.08	18.17	30.05	8.09	11.88	19.97
Multi-para	5.66	9.74	18.17	4.08	8.43	12.51
**Number of living children**						
0	13.84	13.86	45.38	0.02	31.52	31.54
1–5	6.76	12.08	22.56	5.32	10.48	15.8
≥ 6	4.45	7.56	12.06	3.11	4.5	7.61

ANC = Antenatal care, Others = Catholic and traditional followers

### Decomposition analysis

The decomposition analysis model has been taken into account the differences in the characteristics (compositional factors) and the differences due to the effect of characteristics. Only 14% of the overall change in timely first antenatal booking pregnant among women who gave birth five years before the survey was due to the differences in characteristics. Among the compositional factors, a very significant contribution to the change was due to their place of residence, education and occupation status of women.

An increase in the composition of women who lived in rural areas showed a significant contribution to the increase in timely first antenatal booking. Similarly, the increase in the composition of women who have learned primary and above showed a significant contribution to the increase in timely first antenatal booking as compared to the counterpart. Another compositional factor was the occupation status of women. An increase in the proportion of women who had occupations also affects timely first antenatal booking among pregnant women who gave birth five years before the survey positively. Even if compositional changes were too small, an increase in the composition of women who lived in Somali, SNNP and Dire Dawa also positively boost timely first antenatal booking among pregnant women who gave birth five years before the survey. But, the reverse was true for certain regions (Afar, Oromia and Benishangul Gumz) which had a negative impact. Lastly, population compositional change in protestant followers and those women who had one or more living children also play on the reduction of timely first antenatal booking (Table 3).

**Table 3:** Decomposition for changes in timely first antenatal care booking among pregnant women who gave birth in Ethiopia, 2005 to 2016 EDHS.

Category	Difference due to characteristics (E)			Difference due to coefficients (C)		
	Coefficient	Percent	P-value	Coefficient	Percent	P-value
**Age of women**						
15–19	^1^					
20–24	0.00038	0.274	0.322	-0.01037	-7.3865	0.129
25–29	0.000053	0.0376	0.946	-0.00742	-5.2838	0.442
30–34	-0.000367	-0.262	0.760	-0.00486	-3.4645	0.519
35–39	-0.000114	-0.0812	0.553	-0.00246	-1.7545	0.704
40–44	-0.000018	-0.013	0.966	0.00064	0.45681	0.861
45–49	-0.000251	-0.1788	0.776	0.0022	1.548	0.354
**Age at first marriage**						
<18	^1^					
≥18	-0.002543	-1.812	0.072	-0.00753	-5.3637	0.091
**Marital status**						
Not married	^1^					
Married	0.00011	0.0751	0.791	-0.0146	-10.399	0. 545
**Religion**						
Orthodox	^1^					
Protestant	-0.00234	-1.6668	0.001	0.00153	1.0886	0.713
Muslim	0.00121	0.8653	0.237	0.005623	4.0067	0.349
Others	0.00005	0.0356	0.879	0.0019	1.3625	0.370
**Place of residence**						
Urban	^1^					
Rural	0.0033	2.3515	0.020	0.0412	29.34	0.044
**Educational status of women**						
Not educated	^1^					
Primary	0.0084	6.0016	0.000	0.00063	0.4523	0.811
Secondary	0.0012	0.845	0.000	-0.00037	-0.267	0.708
Higher and +	0.00304	2.1642	0.004	-0.00007	-0.0486	0.755
**Occupation status of women**						
Not working	^1^					
Working	0.0045	3.1934	0.044	0.00365	2.6017	0.384
**Sex of household head**						
Female	^1^					
Male	0.00034	0.242	0.546	0.038	27.012	0.014
**Region**						
Tigray	^1^					
Afar	-0.000023	-0.00155	0.000	-0.000142	-0.101	0.662
Amhara	0.00154	1.098	0.080	-0.00376	-2.677	0.483
Oromia	-0.0059	-4.2016	0.000	-0.025	-17.899	0.004
Somali	0.000655	0.4666	0.000	-0.00154	-1.0951	0.277
Benishangul Gumz	-0.00017	-0.121	0.000	-0.000298	-0.212	0.284
SNNP	0.0013	0.95	0.001	-0.0056	-4.0124	0.311
Gambela	0.0000013	0.00095	0.919	-0.00017	-0.1196	0.059
Harari	0.00000791	0.0056	0.342	0.000022	0.0158	0.680
Addis Ababa	0.00036	0.2597	0.166	-0.000245	-0.175	0.559
Dire Dawa	0.00015	0.1099	0.000	0.000019	0.01372	0.829
**Wealth index**						
Poorest	^1^					
Poorer	0.000016	0.0112	0.909	-0.00231	-1.6417	0.703
Middle	-0.00036	-0.2543	0.111	-0.0087	-6.1822	0.123
Richer	-0.00061	-0.4315	0.057	-0.00532	-3.792	0.310
Richest	0.00027	0.1932	0.051	-0.0131	-9.3632	0.003
**Pregnancy wanted**						
No	^1^					
Yes	0.00348	2.4805	0.065	0.0148	10.572	0.097
**Distance to a health facility**						
Big problem	^1^					
Not a big problem	0.0031	2.191	0.231	0.002945	2.0984	0.424
**Number of living children**						
0	^1^					
1–5	-0.00089	-0.6343	0.048	-0.0051	-3.6258	0.891
≥ 6	-0.00092	-0.6585	0.009	-0.0126	-8.9853	0.270
**Parity**						
Null-para	^1^					
Multi-para	0.0011	0.7772	0.105	-0.01827	-13.017	0.285
Total	0.02013	14.342	0.000	0.12021	85.658	0.000

Others = Catholic and traditional followers, SNNP = Southern Nation Nationalities and Peoples and ^1^ = Reference

After controlling the effects of compositional factors, about 86% of the change in timely first antenatal booking was due to the difference in the effects of behavior. Factors including the behavior of rural residents, male household headers and those populations who lived in the Oromia region contributed to change in timely first antenatal booking among pregnant women who gave birth five years before the survey. Especially, more than one-half of the increase in timely first antenatal booking was due to the change in the behavior of the population among rural dwellers and male household heads. As compared to Tigray, the behavior of the population who lived in the Oromia region showed a significant contribution to a negative change in timely first antenatal booking. Again, a small change in timely first antenatal booking among pregnant women who gave birth five years before the survey was due to negative effects of behavioral change in populations who belong to the richest wealth quintile (Table 3).

## Discussion

The result indicated that there is a statistically significant change in timely first antenatal booking among pregnant women in Ethiopia from 2005 to 2016 EDHS. The major contributing factors for this change were due to differences in population composition and changes in the behavior of the population. From changes in population composition, an increase in timely first antenatal booking was due to the increase in the composition of women who were primary educated or above. The finding is similar to that of different studies conducted in a different part of Ethiopia [36, 47–49]. A similar result was reported in African countries [41, 50, 51]. It is also consistent with a study conducted in Nepal [52]. This association may be because the education status of women affects the behavior and their understanding in terms of predicting the importance of ANC [53–55].

Another factor that positively influences timely first antenatal booking was the place of residence. The change in the composition of rural residents results, the change in timely first antenatal booking. This finding indicated that population composition change in rural residents was incredibly increased as compared to urban. This may be due to the previous encountered experience of high fertility in the rural population. Moreover, it may be due to the implementation of different governmental strategies like Health Extension Workers (HEWs) that encourage pregnant women to start early. Increase population composition change of women in their occupation also positively influence timely first antenatal booking. The finding of this study is similar to other studies conducted in Ethiopia [28, 38]. It is also congruent with studies conducted outside Ethiopia [56–58]. This may be due to different job opportunities created by local non-governmental and governmental organizations and affirmative actions.

Composition changes in five regions and one administrative town also affect timely first antenatal booking. Especially, increase the composition of the Oromia population results decrease in timely first antenatal booking. This is also supported by the previous study conducted in Wollega, Ethiopia [35]. The other compositional factor was the total number of living children. As the number of living children increased, the probability of timely first antenatal booking was decreased. This statement agrees with a study conducted in Bahir Dar, Ethiopia and Rwanda [17, 59]. But, it is against that of a study conducted in Ghana [58]. The discrepancy may be because of cultural and other contextual differences. The last compositional factor indicated that as the population proportion of protestant follower’s increased, timely first antenatal booking decreased. This finding may be due to the presence of religious believes that may hinder their behavior [60].

Keeping compositional factors constant, the effect of the behavior of the population also plays a great role in the change in timely first antenatal booking. More than half of the change was due to the change in the behavior of rural residents and male household headers. This may be due to an increase in male involvement in every aspect of maternal and child health care which was one of the recent sustainable development goals agenda and increased media exposure. The other behavioral factors were the effects of the behavior of the Oromia population and those women who lived in the richest wealth quintile. This may be because the richest population may trust their wealth and may not fear further expenses after late initiation. Even if the study utilized a large sample size, take in to accounts different sources of changes (endowment and coefficient) and representative to all parts of Ethiopia, it is not without limitations. Firstly, it is prone to recall bias as the survey asked pregnant women who gave birth five years before the survey. Lastly, as it is secondary data analysis, important variables were missed especially health system factors.

## Conclusions

Timely first antenatal booking among pregnant women who gave birth five years before the survey was significantly increased in Ethiopia for the last one decade. The change was due to both changes in population composition and change in behavior related to population. Mostly, change in the composition of women who gave birth five years before the survey in terms of residency, religion, education and occupation status of women were the sources of the change. The majority of the increase in timely first antenatal booking was due to the difference in the coefficient. For instance, the coefficients of rural residents, male household headers and those populations who lived in the Oromia region contributed to change in timely first antenatal booking among pregnant women who gave birth five years before the survey. Interventions targeting male household headers, rural residents and those women who lived in the Oromia region should be emphasized to increase further in timely first antenatal booking. In addition to this, promoting women in terms of education and creating job opportunities could be the other recalled intervention areas.

## List of abbreviations

ANC: Antenatal care
CSA: Central Statistical Agency
DHS: Demographic and Health Survey
EDHS: Ethiopia Demographic and Health Survey
WHO: World Health Organization
